# Shared Characteristics of Intrinsic Connectivity Networks Underlying Interoceptive Awareness and Empathy

**DOI:** 10.3389/fnhum.2020.571070

**Published:** 2020-12-07

**Authors:** Teodora Stoica, Brendan Depue

**Affiliations:** ^1^Department of Psychological and Brain Sciences, University of Louisville, Louisville, KY, United States; ^2^Department of Anatomical Sciences and Neurobiology, University of Louisville, Louisville, KY, United States

**Keywords:** empathy, interoception, fMRI, ICA, resting-state, connectivity, rsBOLD

## Abstract

Awareness of internal bodily sensations (interoceptive awareness; IA) and its connection to complex socioemotional abilities like empathy has been postulated, yet the functional neural circuitry they share remains poorly understood. The present fMRI study employs independent component analysis (ICA) to investigate which empathy facet (Cognitive or Affective) shares resting-state functional connectivity (rsFC) and/or BOLD variability (rsBOLD) with IA. Healthy participants viewed an abstract nonsocial movie demonstrated to evoke strong rsFC in brain networks resembling rest (*InScapes*), and resultant rsFC and rsBOLD data were correlated with self-reported empathy and IA questionnaires. We demonstrate a bidirectional behavioral and neurobiological relationship between empathy and IA, depending on the type of empathy interrogated: Affective empathy and IA share both rsFC and rsBOLD, while Cognitive empathy and IA only share rsBOLD. Specifically, increased rsFC in the right inferior frontal operculum (rIFO) of a larger attention network was associated with increased vicarious experience but decreased awareness of inner body sensations. Furthermore, increased rsBOLD between brain regions of an interoceptive network was related to increased sensitivity to internal sensations along with decreased Affective empathy. Finally, increased rsBOLD between brain regions subserving a mentalizing network related to not only an improved ability to take someone’s perspective, but also a better sense of mind-body interconnectedness. Overall, these findings suggest that the awareness of one’s own internal body changes (IA) is related to the socioemotional ability of feeling and understanding another’s emotional state (empathy) and critically, that this relationship is reflected in the brain’s resting state neuroarchitecture. Methodologically, this work highlights the importance of utilizing rsBOLD as a complementary window alongside rsFC to better understand neurological phenomena. Our results may be beneficial in aiding diagnosis in clinical populations such as autism spectrum disorder (ASD), where participants may be unable to complete tasks or questionnaires due to the severity of their symptoms.

## Introduction

Internal body signals relative to emotion processing has been a topic of long-standing interest (Gurney, 1884; Strack et al., 1988), with more recent evidence highlighting an intriguing bidirectional relationship between sensations that arise internally and emotional phenomena (Cameron, 2001; Damasio, 2005; Lane, 2008; Craig, 2009). A proposed biological basis that may clarify this interplay is interoception, namely—the afferent processing of internal bodily signals that arise from visceral organs (Cacioppo et al., 2000; Cameron, 2001; Johnson, 2001; Wiens, 2005; Craig, 2009). For example, an increased heart rate signals an emotional modulation, indicating that the assessment of one’s own emotions requires interoceptive processes (Lee and Siegle, 2009). Indeed, evidence suggests a consistent relationship between emotional experience and interoception (Critchley et al., 2004; Pollatos et al., 2007; Werner et al., 2009). Also, neuroimaging findings corroborate a substantial overlap between the neural substrates of one’s own emotional and interoceptive processing. This highlights the proposed idea that interoception plays an important role in emotional self-assessment (Damasio et al., 2000; Terasawa et al., 2013; Adolfi et al., 2017; Critchley and Garfinkel, 2017). However, the relationship between signals arising from one’s own body and the emotions of *another* individual is a topic that remains relatively unexplored.

A harmonious social interaction putatively hinges on whether the observer can vicariously feel and understand the mental state of the listener, a socioemotional ability known as empathy (Davis, 1980). Empathy can be further fractioned into two interrelated facets: Affective and Cognitive Empathy. Affective empathy is conceptualized as the automatic process of vicariously experiencing the emotional state of another (Davis, 1980; Baron-Cohen and Wheelwright, 2004), while Cognitive empathy describes the individual’s ability to accurately imagine another person’s perspective (Davis, 1980; Decety and Jackson, 2004; Lawrence et al., 2006). The two facets of empathy exist on a continuum. While Affective empathy requires the empathizer to represent both “self” and “other,” Cognitive empathy requires a marked “self” and “other” distinction to successfully imagine a different perspective from one’s own (Steinbeis, 2016).

One popular interpretation of such a “shared representation” (Decety and Sommerville, 2003) posits that we represent others’ experiences in terms of self-experience, which may explain why interoceptive awareness (IA; processed internal sensations part of conscious awareness), plays such a crucial role in social encounters (Cameron, 2001; Khalsa et al., 2018). Indeed, a substantial amount of evidence points to IA influencing the degree to which an individual experiences their emotions (Barrett et al., 2004; Wiens, 2005). For instance, those with high IA report heightened emotional arousal (Wiens, 2005; Pollatos and Schandry, 2008; Dunn et al., 2010), which suggests better IA could lead to greater Affective empathy due to the fact the shared emotion is more intense. Besides, increased IA has also been tied to decreased susceptibility to body ownership illusions (Tsakiris et al., 2011; Tajadura-Jiménez and Tsakiris, 2014), suggesting a clearer divergence between “self” and “other” which may positively relate to Cognitive Empathy.

Current neuroimaging evidence indeed suggests that the neural substrates of empathy overlap with those involved in self-experience (Wicker et al., 2003; Keysers et al., 2004; Iacoboni, 2005; Jackson et al., 2005), supporting the theory that the brain represents others’ experiences in terms of the experiences of the self (Decety and Sommerville, 2003). For instance, in the Jabbi et al. (2007) study, activation of the anterior insula (AI) and inferior frontal operculum (IFO) was observed in both the observer and experiencer during aversive taste stimuli. Similarly, observing others’ pain has been found to robustly activate the AI and anterior cingulate cortex (ACC), regions associated with one’s own pain (Singer et al., 2004; Jackson et al., 2005; Lieberman and Eisenberger, 2009).

However, in the “shared representation” context, it is unclear which brain regions underlying a *specific* aspect of empathy contribute to IA. This may be due to empathy’s facets activating interacting, but only partially overlapping, neural bases (Fan et al., 2011). Affective empathy primarily elicits activations from regions implicated in rapid and prioritized processing of emotion signals, including the amygdala, hypothalamus, orbitofrontal cortex (OFC), and AI (Decety et al., 2013). By comparison, Cognitive Empathy, which shares similar neural networks with mentalizing and Theory of Mind (TOM; Pardini and Nichelli, 2009) additionally involves the superior temporal sulcus (STS), temporoparietal junction (TPJ), fusiform gyrus (FG), and medial prefrontal cortex (mPFC; Saxe and Powell, 2006). Thus, it is plausible to theorize that IA may share neural bases with Affective empathy within the AI and amygdala, and with Cognitive empathy within the PFC. A better understanding of whether there is a disassociation between these constructs concerning IA could therefore refine and extend the “shared representation” hypothesis.

Although no studies have explored the neural intersection of IA and empathy’s two facets, one recent meta-analysis did investigate convergent areas of activation between IA, emotion, and social cognition (Adolfi et al., 2017). The results for the three domains converged in the AI, amygdala, right inferior frontal gyrus (rIFG), basal ganglia, and medial anterior temporal lobe (mATLc), ascribing particular importance to the fronto-temporo-insular nodes (Adolfi et al., 2017). The authors conclude co-activation of these regions may result in an evaluative association of the internal milieu, and in combination with external cues, leads to complex social cognition (Adolfi et al., 2017). However, only partial insight can be gleaned from these results in connection to the present study. The authors of the Adolfi et al.’s (2017) study describe the complex domain of “social cognition” simply in terms of TOM (the attribution of mental states to oneself and others; Baron-Cohen et al., 2001). TOM only takes into account the Cognitive and not Affective facet of empathy, and according to the Shamay-Tsoory et al. (2010) model, both are required for intact empathic processing. Therefore, within the framework of the “shared representation” hypothesis, this meta-analysis only offers a limited glimpse into how IA and empathy’s facets are neurologically related.

Nevertheless, this activation-based meta-analysis revealed several key brain regions known to play a role within a distributed socioemotional network. Scant functional connectivity (FC) data exists directly addressing how these regions communicate and how their communication could result in representing others’ experiences in terms of the experiences of the self (Decety and Sommerville, 2003). Thus far, one study investigating deficits in a patient with depersonalization disorder (body self-awareness disruption) employed graph-theory analyses during an empathic task and demonstrated impaired Affective empathy and IA related to changes in an interoceptive-emotional network, specifically in the AI, ACC, and somatosensory cortex (Sedeño et al., 2014). Although germane, the study only supports an association between these domains during active, task-relevant network configurations. However, if the brain uses the ‘self’ as a blueprint for understanding others’ emotional experiences as proposed by Decety and Sommerville (2003), it stands to reason that the brain’s intrinsic connectivity networks (ICNs) during resting-state (rsfMRI) already contain the information necessary for task-based expression.

Several studies corroborate this assumption. Recently Tavor et al. (2016) applied computational models showing that resting-state functional connectivity (rsFC) alone is sufficient to predict individual variability in task maps and that this pattern of intrinsic connectivity can be predictive of a subject’s identity, similar to a fingerprint (Finn et al., 2015). Importantly, Bilevicius et al. (2018) illustrated that empathy scores were correlated with different patterns of rsFC in the default mode network (DMN), salience network (SN), and left and right central executive networks (CEN). Similarly, Cox et al. (2012) showed that relative empathic ability (REA) is reflected in the brain’s rsFC. Last, Christov-Moore et al. (2020) utilized machine learning to demonstrate rsFC patterns within the resonance, and CEN networks can predict trait empathic concern (EC). No evidence regarding trait IA within rsfMRI exists thus far, but studies point to a large-scale brain system supporting interoception comprising the DMN and SN (Kleckner et al., 2017). Therefore, it is possible to hypothesize that empathy and IA could share rsFC within the DMN or SN, supporting the “shared representation” hypothesis through rsFC data.

In addition to rsFC, blood oxygenation level-dependent (BOLD) variability is an often discounted neuroimaging measurement that may offer complementary information regarding network function and organization. What BOLD variability represents has been unclear, but recent neuroimaging advances suggest it may reflect network coherence throughout the cortex, and therefore a complementary reflection of FC (Fox, 2005; Mišíc et al., 2011; Vakorin et al., 2011b). Although BOLD variability is often ignored because it has been attributed to various confounds that are deliberately minimized (in the name of improving signal-to-noise ratios; Garrett et al., 2013), several areas of neuroscience research have examined the properties and unique functionality of variance, and suggest that by considering rather than ignoring variance, our ability to understand and predict neural phenomena can improve dramatically (Stein et al., 2005; MacDonald et al., 2006; Faisal et al., 2008). Recent theories consider high BOLD variability necessary for the neural system’s adaptability, efficiency, and cognitive performance (McIntosh et al., 2008; Garrett et al., 2010, 2013; Vakorin et al., 2011a,b; Dai et al., 2016). Specifically, according to the coordination dynamics theory, networks demonstrating increased BOLD variability can flexibly shift through integrative and segregative configurations, maintaining the neural system in balance (Tognoli and Scott Kelso, 2014). In the same way, rsFC is used to predict task performance in individual subjects (Finn et al., 2015), resting-state BOLD variability (rsBOLD) is used to show that the subject- and task-specific BOLD variability signature is stable and persistent across time (Gaut et al., 2019). rsBOLD variability has been used in clinical populations (Scarapicchia et al., 2018; Good et al., 2020; Kumral et al., 2020; Zhang et al., 2020) and in healthy populations to investigate brain maturation trajectories (Nomi et al., 2017) and degree of cognitive flexibility (Armbruster-Genç et al., 2016). Although no studies in a healthy sample have yet explored rsBOLD variability concerning trait empathy or IA, this inquiry could shed light on how networks underlying these constructs communicate. For instance, increased rsBOLD variability in SN and/or DMN concerning empathy and IA could putatively be related to effective switching between “self ” and “other,” leading to successful empathizing.

Therefore, the present study employs a data-driven approach to explore rsFC and rsBOLD variability related to brain networks underlying Cognitive, Affective empathy, and IA. Specifically, we aim to understand whether Cognitive and/or Affective empathy as measured by self-report questionnaires share rsFC and/or rsBOLD variability with IA self-report measures during resting state in healthy adults. We hypothesize based on previous literature that: (1) affective empathy will share rsFC and/or rsBOLD with IA within an SN network, specifically the amygdala, AI, and IFO, given their involvement in the processing of emotion experienced in oneself and vicariously for others (Singer et al., 2004); while (2) cognitive empathy will share rsFC/ rsBOLD variability with IA within a mentalizing network, specifically in the rTPJ and precuneus, as these regions are posited to underlie explicit mentalizing (Kovács et al., 2014; Hyde et al., 2015; Bardi et al., 2017; Naughtin et al., 2017).

## Materials and Methods

### Participants and Procedure

Twenty-six healthy young adults (m = 21.85 years old/16 females) without a reported history of neurological or psychiatric disorders were recruited for this study (Table 1). This sample size provided 80% power for detecting an effect (*r*) as small as 0.50. All participants were right-handed and had normal or corrected-to-normal vision and hearing. Participants were recruited through on-campus flyers. All participants were paid $20 for their participation. Experimental protocols were approved by the University of Louisville’s Institutional Review Board before data collection and written informed consent was obtained from each participant before experimental sessions. The study took part in two separate days. On the first day, participants visited the lab to be briefed on the MRI protocol, fill out consent forms, and behavioral assessments. On the second day, participants completed the rsfMRI scan at the University of Louisville, School of Medicine.

**Table 1:** Sociodemographic characteristics of participants.

	Frequency	Min	Max	%
Gender	10	Male		38.5
	16	Female		61.5
	*Total: 26*			
Ethnicity	1	Hispanic/Latino		3.8
	24	Not Hispanic/Latino		92.3
	1	Unknown		3.8
Race	22	White		84
	1	African American		4
	2	Asian		8
	1	More than 1 Race		4
				Mean
Age		18	31	21.8
Education (years)		12	21	14.2

### Stimulus: “Inscapes” Paradigm

**Figure 1:**
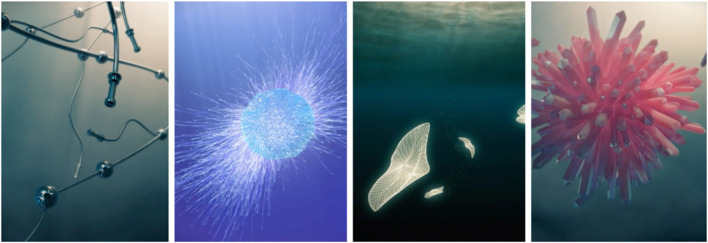
Still-shots from the InScapes movie.

Resting-state scans have previously been utilized to investigate IA (Kuehn et al., 2016; Chong et al., 2017), and naturalistic stimuli like movies have been shown to elicit robust neurobiological emotional responses such as empathy (Westermann et al., 1996; Borja Jimenez et al., 2020). However, since the scope of this study was an inquiry into the relationship *between* IA and empathy, we chose to utilize an intermediate stimulus called “*Inscapes*.” The 7 min abstract, the nonsocial movie titled *Inscapes* has previously been demonstrated to evoke strong connectivity in networks that resemble rest more than those exhibited during conventional movies (Vanderwal et al., 2015). The movie features a series of technological-looking abstract shapes (Figure 1). Participants were told to keep their eyes open and relax while watching and listening to the movie. The stimulus was displayed using E-prime on an Invivo Esys LCD TV monitor at the back of the scanner bore, which was viewed by participants through a mirror on the head-coil. The video is freely available for download from HeadSpace Studios.

### Behavioral Assessments

#### Empathy Questionnaire—Interpersonal Reactivity Index (IRI)

Affective and Cognitive empathy was assessed using the Interpersonal Reactivity Index (IRI; Davis, 1983). The IRI consists of 28 items rated on a 5-point scale with the anchors: “does not describe me well” to “describes me very well.” The items are arranged into four subscales with seven items. Each subscale measures a distinct component of empathy: EC (feelings of compassion and concern for others); personal distress (PD; feelings of anxiety and discomfort that result from observing another person’s negative experience); perspective taking (PT; the ability to adopt the perspectives of other people and see things from their point of view); and fantasy subscale (FS; the tendency to identify with characters in movies, books, or other fictional situations; Davis, 1983). Affective empathy, the ability to infer an agent’s feelings or emotions, was derived from summing the EC and PD subscales. Cognitive empathy, the ability to infer an agent’s beliefs or thoughts, was derived from summing the FS and PT subscales. Total empathy was derived from aggregating Affective and Cognitive empathy scores. All scores were standardized by applying a *z*-score transformation and later compared with the MAIA assessment and its subscales (see below) in subsequent analyses. Correlations between behavioral measurements were conducted with the Statistical Package for Social Sciences (Version 25.0.0; SPSS Inc.), and corrected for age, gender, and multiple comparisons using false discovery rate *p* < 0.05 (FDR; Benjamini and Hochberg, 1995).

#### Multidimensional Assessment of Interoceptive Awareness (MAIA)

The Multidimensional Assessment of Interoceptive Awareness (MAIA) is a 32-item instrument assessing IA: “the conscious perception of sensations from inside the body that creates the sense of the physiological condition of the body, such as heartbeat, respiration, satiety, and the autonomic nervous system sensations related to emotions” (Mehling et al., 2012). Each statement is rated from 0 (never) to 5 (always) in terms of how often it applies to the participant generally in daily life. The statements are then separated into eight subscales: Noticing, Non-Distracting, Not-Worrying, Attention-Regulation, Emotional Awareness, Self-Regulation, Body Listening, and Trusting, which are in turn aggregated into five overall scales used in the present study: Awareness of Body Sensations (Noticing); Emotional Reaction and Attentional Response to Sensations (Not-Distracting, Not-Worrying); Capacity to Regulate Attention (Attention Regulation), Awareness of Mind-Body Integration (Emotional Awareness, Self-Regulation, Body Listening) and Trusting Body Sensations (Trusting). A total Interoceptive Score (MAIA Total) was derived by summing all the aggregate scales. All scores were standardized by applying a *z*-score transformation and later compared with the IRI and its subscales (see above) in subsequent analyses. Correlations between behavioral measurements were conducted with the Statistical Package for Social Sciences (Version 25.0.0; SPSS Inc.), and corrected for age, gender, and multiple comparisons using false discovery rate *p* < 0.05 (FDR; Benjamini and Hochberg, 1995).

### MRI Data Acquisition and Preprocessing

All structural MRI images were acquired using a Siemens 3-T Skyra MR scanner. A 20-channel head coil was used for radiofrequency reception. Participants were given earplugs to reduce scanner noise and were additionally given headphones to receive instructions. Foam padding was added to limit motion if additional room remained within the head coil, and a piece of folded tape was placed over the participant’s forehead as a reminder to remain still throughout the scan. Structural images were obtained *via* a T1-weighted magnetization-prepared rapid gradient-echo sequence (MPRAGE) in 208 sagittal slices. Imaging parameters were as follows: echo time (TE) = 2.26 ms, repetition time (TR) = 1,700 ms, flip angle = 9.0°, field of view (FoV) = 204 mm, and voxel size = 0.8 × 0.8 × 0.8 mm. Scan parameters were consistent for all imaging sessions associated with this study. Functional BOLD images were collected using a multi-band acceleration factor of 3. Two-hundred ten volumes were collected. Imaging parameters were as follows: TE = 29 ms; TR = 2,000 ms; flip angle = 62°; FoV = 250 mm; isotropic voxel size = 2.0 mm^3^; 78 interleaved slices, GRAPPA on, Partial Fourier 7/8. Slices were oriented obliquely along the AC-PC line.

All analyses were conducted using the CONN toolbox 19.c (Whitfield-Gabrieli and Nieto-Castanon, 2012) based on SPM12 (Penny et al., 2007) in the 2017 version of MATLAB. Spatial preprocessing in the CONN toolbox included the functional realignment and unwarping; slice-timing correction; outlier identification; direct segmentation and normalization; and functional smoothing (6 mm FWHM Gaussian filter) using SPM12 default parameter settings. Detailed steps can be found in CONN documentation (Whitfield-Gabrieli and Nieto-Castanon, 2012), but briefly: (1) all scans were coregistered and resampled to a reference image (first scan of the first session) using b-spline interpolation; (2) temporal misalignment between different slices of the functional data, introduced by the sequential nature of the fMRI acquisition protocol, was corrected using SPM12 slice-timing correction (STC) procedure (Henson et al., 1999) where the functional data was time-shifted and resampled using sinc-interpolation to match the time in the middle of each TA (acquisition time); (3) potential outlier scans were identified from the observed global BOLD signal and the amount of subject-motion in the scanner using the ART toolbox, and no outliers were identified; (4) functional and anatomical data were normalized into standard MNI space and segmented into gray matter, white matter, and CSF tissue classes using SPM12 unified segmentation and normalization procedure (Ashburner and Friston, 2005); and (5) last, functional data was smoothed using spatial convolution with a Gaussian kernel of 6 mm full width half maximum (FWHM). Next, a 0.01–0.10 Hz temporal band-pass filter standard for resting-state connectivity analyses was applied to the time series (Nieto-Castanon, 2015) as part of CONN’s default denoising step. In sum, detrending, outlier censoring, motion regression, and CompCor correction were performed simultaneously in a single first-level regression model, followed by band-pass filtering. These corrections yielded a residual BOLD time course at each voxel that was used for subsequent analyses.

### Neuroimaging Analysis

#### Network Connectivity

A measure of network connectivity during resting state (i.e., networks of functionally connected brain regions) was derived from group-level independent component analysis (ICA) using the CONN toolbox. We then investigated how network connectivity may be associated with the IRI and MAIA scores. This involved the application of the fast ICA algorithm to volumes concatenated across subject and resting-state condition to identify independent components (ICs) and back-projection of these components to individual subjects, resulting in maps of regression coefficients representing connectivity between the network and every voxel in the brain (see Calhoun et al., 2001 for details). We chose 40 ICs due to research suggesting ICA results are only affected by the number of ICs when the number is smaller than the number of source signals (Ma et al., 2007), in addition to assuring coverage of a majority of the signal variance. Out of the 40 resultant ICs, noise components were identified through visual inspection by authors (TS and BD; e.g., components largely overlapping CSF), resulting in the exclusion of 11 ICs from further consideration. Out of the remaining 29 ICs, eight networks were identified using the spatial overlap of suprathreshold areas (Dice coefficient; Rombouts et al., 1998), based on CONN’s default network atlas with ROIs characterizing an extended set of eight classical brain networks: Default Mode Network (four ROIs), Sensorimotor (two ROIs), Visual (four ROIs), Salience/Cingulo-Opercular (seven ROIs), Dorsal Attention (four ROIs), Frontoparietal/Central Executive (four ROIs), Language (four ROIs), Cerebellar (two ROIs; all ROIs defined from CONN’s ICA analyses of HCP dataset/497 subjects; Nieto-Castanon, 2014). We next selected six ICs that exhibited networks that have been previously associated with IA and/or empathy: Frontoparietal/Central Executive, Default Mode Network, Sensorimotor, Cerebellar, Salience/Cingulo-Opercular and Dorsal Attention (Bernhardt and Singer, 2012; Takeuchi et al., 2013; Stern et al., 2017; Kleckner et al., 2017; Bilevicius et al., 2018; Jauniaux et al., 2019). The chosen six ICs were subsequently entered in multiple regressions with the IRI and MAIA subscales, aggregate, and Total scores. Type I error was controlled using cluster-size-based false discovery rate (FDR) correction [*p* < 0.05, voxel thresholded at *p* < 0.001 (Worsley et al., 1996), within each analysis], and FDR-corrected (pFDR < 0.03) *across* networks. Furthermore, results were corrected for age and gender. All coordinates reported below refer to peak activations in anatomical MNI space.

#### Network Variability

To assess network variability (i.e., network coherence), and its relationship to either empathy or IA, we regressed each IC’s network variability (calculated in CONN as SD of each IC’s BOLD time-series: SD_BOLD_; Nieto-Castanon, 2020) with the IRI and MAIA subscales, aggregate and Total scores. Type I error was controlled (within each analysis) with FDR-corrected significance thresholds (pFDR < 0.03) across networks and corrected for age and gender.

## Results

### Behavioral Results

To establish the relationship between empathy and IA, all IRI and MAIA subscales, aggregate, and Total scores were correlated (Table 2).

**Table 2:** Intercorrelations between Interpersonal Reactivity Index (IRI) and Multidimensional Assessment of Interoceptive Awareness (MAIA) questionnaires and descriptive statistics.

	1	2	3	4	5	6	7	8	9	10	11	12	Mean	STD
IRI Total (1)	−												75.79	11.12
IRI Affective Empathy (2)	0.60	−	−										31.84	6.3
IRI Personal Distress (3)	0.11	0.79	−										10.24	5.61
IRI Empathic Concern (4)	0.80	0.48	−0.17	−									21.6	3.85
IRI Cognitive Empathy (5)	0.83	0.05	−0.41	0.67	−								44	8.52
IRI Perspective Taking (6)	0.58	−0.15	−0.52	0.50	0.83	−							21.64	4.97
IRI Fantasy (7)	0.80	0.22	−0.18	0.61	0.84	0.39	−						22.36	5.25
MAIA Total (8)	−0.23	−0.64*	−0.66*	−0.08	0.16	0.31	−0.04	−					23.28	4.36
MAIA Awareness of Body Sensations (9)	−0.30	−0.36	−0.19	−0.29	−0.12	−0.17	−0.03	0.54	−				2.98	0.81
MAIA Emotional Reaction (10)	−0.33	−0.23	−0.21	−0.08	−0.25	−0.11	−0.31	0.37	−0.03	−			5.65	1.26
MAIA Capacity to Regulate Attention (11)	−0.33	−0.83*	−0.74*	−0.27	0.17	0.32	−0.03	0.79	0.48	0.24	−		3.05	0.94
MAIA Awareness of Mind Body Integration (12)	0.09	−0.34	−0.49*	0.16	0.35	0.41*	0.18	0.82	0.37	−0.05	0.50	-	8.12	2.46
MAIA Trusting Body Sensations (13)	−0.25	−0.55*	−0.53*	−0.12	0.08	0.31	−0.18	0.71	0.21	0.32	0.53	0.40	3.48	1.08

Intercorrelations between IRI and MAIA subscales and descriptive statistics (same subscale correlations not flagged for significance). *Represents correlations statistically significant after multiple comparison correction. Uncorrected Mean and STD reported. Significant values are represented in bold.

#### Relationship Between Affective Empathy and Interoceptive Awareness

Excluding same-subscale correlations, negative relationships were observed between Affective empathy (EC + PD) and the following MAIA subscales: Capacity to Regulate Attention (*r*_(26)_ = −0.83, *p* < 0.01), Trusting Body Sensations (*r*_(26)_ = −0.55, *p* < 0.01) and MAIA Total (*r*_(26)_ = −0.64, *p* < 0.01). Similarly, we observed a negative relationship between the PD subscale and the Capacity to Regulate Attention (*r*_(26)_ = −0.74, *p* < 0.01), Awareness of Mind Body Integration (*r*_(26)_ = −0.49, *p* < 0.01), Trusting Body Sensations (*r*_(26)_ = −0.53, *p* < 0.01) and MAIA Total (*r*_(26)_ = −0.66, *p* < 0.01). Therefore, we report a negative relationship between Affective empathy and IA (most influenced by the PD scale, since EC exhibited no significant relationship within our sample). Although we do report subscale results above, we only refer to this scale as Affective empathy henceforth.

#### Relationship Between Cognitive Empathy and Interoceptive Awareness

We observed a positive relationship between Cognitive Empathy (PT + Fantasy) and the Awareness of Mind-Body Integration subscale, *r*_(26)_ = 0.35, *p* = 0.06, although it did not survive multiple comparison correction. Also, we observed a significant positive relationship between the IRI PT subscale, and the MAIA Awareness of Mind-Body Integration subscale, *r*_(26)_ = 0.41, *p* < 0.01. Therefore, we report a positive relationship between Cognitive empathy and IA (most influenced by the PT subscale, since Fantasy exhibited no significant relationship within our sample). Although we do report subscale results above, we only refer to this scale as Cognitive empathy henceforth.

Therefore, taken together, our behavioral results show a bidirectional relationship between empathy and IA, depending on the facet of empathy interrogated and mainly driven by the subscales of PD (Affective Empathy) and PT (Cognitive Empathy).

### Functional Connectivity Results

#### Network Connectivity

**Figure 2:**
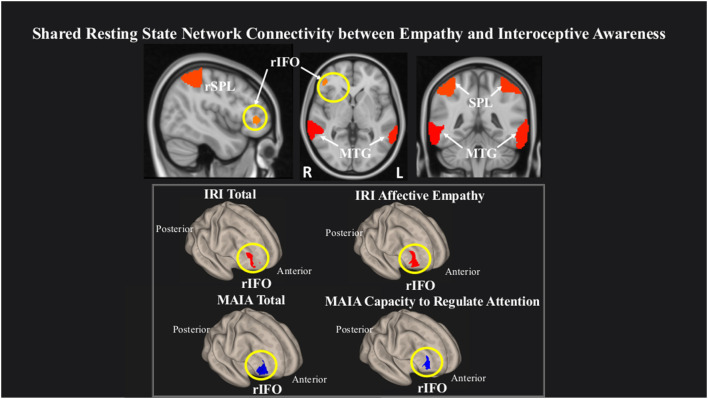
Greater connectivity in the right inferior frontal operculum (rIFO) is associated with lower total IA and greater Affective empathy. Statistical maps are FDR-corrected within the network at cluster-based *p* < 0.05, after voxel threshold at *p* < 0.001, and further corrected for age, gender, and multiple comparisons across components using FDR.

We observed that within a network comprising right inferior frontal operculum (rIFO), bilateral superior parietal lobule (SPL), and bilateral middle temporal gyrus (MTG), greater rsFC in the rIFO was associated with greater overall empathy as measured by the IRI Total, and with greater IRI Affective empathy. Conversely, lower rsFC in the rIFO was associated with increased overall IA as measured by the MAIA Total and increased MAIA Capacity to Regulate Attention (Figure 2, Table 3).

**Table 3:** Network connectivity statistics.

Questionnaire subscale	Region	Laterality	Peak cluster (*X*, *Y*, *Z*)	Size	*p*-FDR	Effect size	90% CI
MAIA total	IFO	R	46, 52, 2	289	0.02	2.7	2, 3.25
Capacity to regulate attention	IFO	R	46, 48, 14	407	0.003	2.8	2, 3.25
IRI total	IFO	R	36, 56, 6	207	0.03	2.7	1.5, 3
Affective empathy	IFO	R	40, 48, −4	284	0.02	2.8	2, 3.5

*Connectivity statistics of rIFO cluster within the network associated with lower total IA, MAIA Capacity to Regulate Attention, and greater total Empathy and IRI Affective empathy*.

#### Network Variability

**Figure 3:**
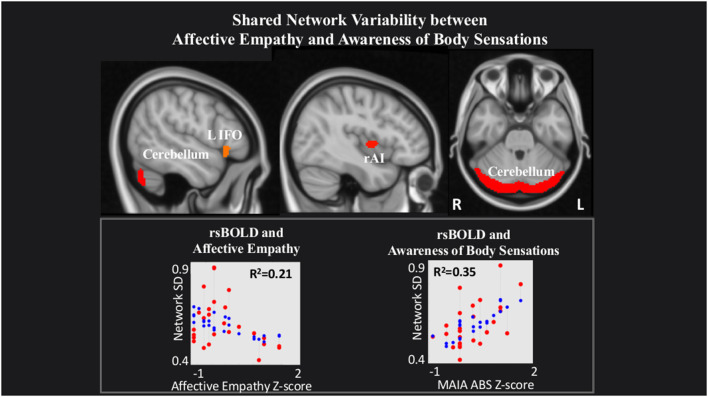
Shared network variability between Affective empathy and MAIA Awareness of Body Sensations. Statistical maps are FDR-corrected within the network at cluster-based *p* < 0.05, after voxel threshold at *p* < 0.001, and further corrected for age, gender, and multiple comparisons across components using FDR. *Note:* the red dots in the graph represent the observed correlation between the standard deviation of the individual network’s BOLD time-series (SD_BOLD_) and each subjects’ behavioral measure. The blue dots represent the predicted values of the statistical model. The *R*^2^ value represents the variance explained resulting from the regression between SD_BOLD_ and behavioral variables of interest.

rsBOLD analyses revealed that within a network comprising left IFO (L IFO), Cerebellum, and rAI, IRI Affective Empathy was negatively related to rsBOLD of the network (*T*_(22)_ = −2.23, *p* = 0.03), while conversely, MAIA Awareness of Body Sensations was positively related (*T*_(22)_ = 3.34, *p* = 0.002; Figure 3).

**Figure 4:**
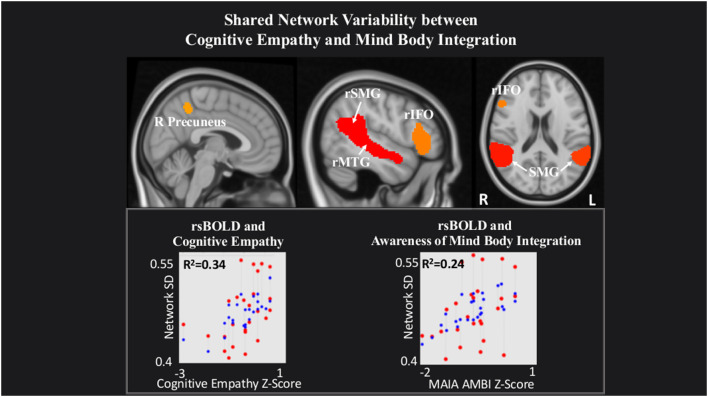
Shared network variability between Cognitive empathy and MAIA Mind-Body Integration statistical maps are FDR-corrected within the network at cluster-based *p* < 0.05, after the voxel threshold at *p* < 0.001, and further corrected for age, gender, and multiple comparisons across components using FDR. *Note:* the red dots in the graph represent the observed correlation between the standard deviation of the individual network’s BOLD time-series (SD_BOLD_) and each subjects’ behavioral measure. The blue dots represent the predicted values of the statistical model. The *R*^2^ value represents the variance explained resulting from the regression between SD_BOLD_ and behavioral variables of interest.

Last, within a network comprising right Precuneus, rMTG, bilateral supramarginal gyrus (SMG) and rIFO, IRI Cognitive Empathy (*T*_(22)_ = 2.56, *p* = 0.02) and MAIA Mind-Body Integration (*T*_(22)_ = 2.52, *p* = 0.02) were positively related to rsBOLD (Figure 4).

## Discussion

Empathy and IA are crucial to meaningful social exchanges. As these two constructs interact, a “shared representation” is created as one’s own internal state is utilized to understand the emotional experiences of others (Decety and Sommerville, 2003). However, it is not yet clear whether a specific aspect of empathy (Affective or Cognitive) interfaces with IA. Our resting-state fMRI study employed ICA to investigate which empathy facet shares rsFC and/or rsBOLD with IA while healthy adults viewed naturalistic stimuli. We observed a bidirectional behavioral relationship between empathy and IA, whereby Affective empathy and IA were negatively related, and Cognitive empathy and IA were positively related. This bidirectional link is mirrored in the neuroimaging results, such that Affective empathy and IA were inversely related to increased rsFC within the rIFO, and also inversely related to rsBOLD; whereas Cognitive empathy and IA showed only a positive relationship with rsBOLD. Together, these results suggest a double disassociation between empathy and IA depending on the type of empathy interrogated, which is reflected in the brain network’s intrinsic connectivity and variability patterns.

Behaviorally, we observed a negative relationship between the PD subscale of the Affective empathy aggregate IRI scale and the total MAIA score, Capacity to Regulate Attention, and Trusting Body Sensations subscales. The Capacity to Regulate Attention subscale pertains to various ways of controlling one’s attention towards bodily sensations, as part of an active regulatory process; while Trusting Body Sensations reflects the extent to which one views awareness of bodily sensations as helpful for decision making (Mehling et al., 2012). The EC subscale of Affective empathy exhibited no significant relationship, implying that PD is the dominant subscale of the Affective empathy aggregate scale when relating to the MAIA within this sample. This distinction is important, considering that previous data suggest EC motivates individuals to pay attention *to others’* emotions to try to comfort them, while conversely, PD drives attention *away from others* to reduce the aversive effects for oneself, perhaps as a form of emotion regulation (Zaki et al., 2014). Indeed, Decety and Jackson (2004) proposed that PD may arise from the failure of applying sufficient self-regulatory control over the shared emotional state. In line with previous studies, we report an inverse relationship between PD and an attention regulation measure—MAIA’s Capacity to Regulate Attention subscale. Together with the Trusting Body Sensations subscale, our findings suggest the increased ability to regulate internal attention and rely on this discrete information may be linked to a decrease in the discomfort experienced while witnessing another’s distress.

Furthermore, we found a positive relationship between the PT subscale of the Cognitive empathy aggregate IRI scale and the Awareness of Mind-Body Integration of the MAIA.This MAIA subscale represents the integration of several higher-level cognitive processes necessary for socially relevant goal-directed behavior (i.e., executive functions; Pribram, 1973) including emotional awareness, self-regulation of emotions, and the ability to feel a sense of an embodied self, that is—“a sense of the interconnectedness of mental, emotional, and physical processes as opposed to a disembodied sense of alienation from one’s body” (Mehling et al., 2012). Thus, our results support previous findings suggesting Cognitive empathy, and in particular, PT is related to a wide array of executive function skills such as working memory, inhibitory control, and cognitive flexibility (Aliakbari et al., 2013; Healey and Grossman, 2018; Yan et al., 2020). Taken together with the aforementioned negative relationship between PD and IA, these behavioral results suggest a bidirectional “shared representation” between empathy and IA, contingent on the type of empathy interrogated. To wit, directing attention towards internal bodily sensations may relieve vicarious emotional pain but flexibly employing cognitive-control skills may increase the ability to take the perspective of another.

Our rsFC results provide further support for this inverse relationship. Within a network of brain regions previously shown to underlie attentional processing [superior parietal lobule (SPL), medial temporal gyrus (MTG), and rIFO (Perrett et al., 1982, 1985, 1992; Corbetta and Shulman, 2002; Wu et al., 2016), we observed that increased rsFC in the rIFO was associated with increased overall empathy (total IRI score) and the Affective empathy aggregate scale on one hand, but reduced overall IA (total MAIA score) and Capacity to Regulate Attention on the other hand. Previous studies investigating both personal (Johnson, 2001; Critchley, 2005; Damasio, 2005; Gray et al., 2007; Craig, 2009) and vicarious emotional experience (Singer et al., 2004; Jabbi et al., 2007) show the consistent activation of the AI and frontal operculum, and therefore the IFO is thought of as a continuum between these two structures (Wicker et al., 2003; Jabbi et al., 2007). Because Affective empathy was driven by the PD subscale within this sample, increased rsFC within the rIFO in the present study may relate to intensified personal suffering from witnessing another’s distress, but decreased awareness of one’s own body sensations, perhaps due to allocating attention externally (for example, away from self and toward other’s distress). In line with previous activation-based results (Ernst et al., 2013; Adolfi et al., 2017), our findings refine the “shared representation” hypothesis (Decety and Sommerville, 2003) by showing rsFC overlap of IA and Affective empathy in this region, and extend previous results by providing rsFC evidence of a double dissociation between empathy and IA.

Our rsBOLD results offer a complementary perspective that further supports this bidirectional relationship. We observed increased scores on the MAIA Awareness of Body Sensations subscale and decreased scores on the Affective empathy scale was associated with increased rsBOLD of brain regions previously shown to underlie processing and integration of visceral information (i.e., Cerebellum, L IFO, L AI; Schmahmann, 2001; Baumann and Mattingley, 2012; Schienle and Scharmüller, 2013; Terasawa et al., 2013; Bogg and Lasecki, 2015; Adamaszek et al., 2017). Despite the prevailing focus on the AI as a hub for processing body sensations (Pollatos et al., 2007; Singer et al., 2009; Terasawa et al., 2013; Kuehn et al., 2016), additional brain regions are also commonly implicated in interoceptive experience. For example, fMRI studies identify the involvement of the IFO and cerebellum, reinforcing the notion that interoceptive processing (and perhaps especially nociceptive information) may occur through multiple neural pathways (Peiffer et al., 2008; Rapps et al., 2008; Garcia-Larrea, 2012). In the same vein, observing distress in others without actually experiencing it may rely on high-order cognitive functions to access minor changes in physical state, as a tool to modulate negative stimulus input (Preckel et al., 2018). The implication of the cerebellum in a shared network underlying both Affective empathy and interoceptive processing is not surprising, since the cerebellum serves as an integral node in the distributed cortical-subcortical neural circuitry supporting an array of sociocognitive operations (Schmahmann and Pandya, 1995). Thus, our rsBOLD findings offer a complementary perspective alongside our rsFC data, and suggest that increased communication between regions of this network relates to increased awareness of internal sensations and perhaps a sense of “self,” but decreased flexibility in integrating emotions arising from witnessing “others”’ distress.

Also, we observed a positive relationship between rsBOLD and the Cognitive empathy scale and the MAIA Awareness of Mind-Body Integration subscale within a network of brain regions previously associated with the process of mentalizing—the precuneus, rIFO, SMG, and MTG (Vogeley et al., 2001; Northoff et al., 2006; Spreng et al., 2009; Mar, 2011; Schurz et al., 2014). Mentalizing signifies the ability to attribute mental states to another individual, allowing the observer to predict intent and direct their behavior appropriately (Premack and Woodruff, 1978; Frith et al., 1991). Researchers agree that mentalizing differs from the vicarious sharing of emotion in its psychological complexity, combining observation, memory, knowledge, and reasoning to provide insight into the thoughts and feelings of others (Decety and Jackson, 2004; van der Heiden et al., 2013). Therefore, its connection with MAIA’s Awareness of Mind-Body Integration scale is not surprising, since both concepts require not only effective experience but also comprehension and integration of another’s particular state of mind within one’s emotional schema. We, therefore, suggest that increased rsBOLD of brain regions underlying a mentalizing network may point to enhanced network flexibility subserving not only a better ability to take another’s perspective, but also an improved sense of interconnectedness between one’s own mind and body.

Last, our data shows an interesting convergence of empathy and IA within the IFO. Research suggests the IFO serves as both a sensory-cognitive integration area and a control node of the ventral attention network (Corbetta and Shulman, 2002; Craig, 2009), conjectured to maintain goal-related information online until a decision is reached (Tops and De Jong, 2006; Tops et al., 2010). Moreover, recent evidence suggests a hemispheric specialization of the IFO related to reactive/proactive goal maintenance (Tops et al., 2010). On one hand, the rIFO may facilitate immediate somatosensory processing and attentional shifting whilst a response is ongoing (reactive; Hampshire et al., 2009; Nelson et al., 2010; Tops et al., 2010; Higo et al., 2011), through its connections to rostral ACC, superior temporal gyrus (STG) and occipital cortex (Cauda et al., 2011). On the other hand, the lIFO may exert top-down control whilst preparing a response (proactive; Tops et al., 2010), through its connections to the dorsolateral prefrontal cortex and bilateral supplementary motor area (Cauda et al., 2011). Taking this evidence into consideration, we speculate the positive association between rsFC within the rIFO and Affective empathy indicates a propensity in the highly empathic individual to shift attention toward salient cues in their environment (for example, another individual in distress). In contrast, the negative relationship between rsFC within the rIFO and IA may indicate an inability to redirect attention toward external salient cues and therefore may lead to increased awareness of one’s internal sensations. Our rsBOLD findings offer complementary evidence regarding the role of the IFO in socio-emotional processes. We show that increased network flexibility within an interoceptive experience network (comprised of lIFO, L AI, cerebellum) is linked to increased Awareness of Body Sensations as well as decreased Affective empathy. We suggest the lIFO plays a crucial part in this network’s ability to modulate attention from one’s internal sensations (i.e., the “self”) to discomfort arising from witnessing the “others” distress, perhaps to plan an appropriate emotional response. In the same vein, we show enhanced network flexibility within a mentalizing network (comprised of rIFO, precuneus, SMG, MTG) is related to both better Cognitive empathy and increased Mind-Body Integration. These relationships may illustrate that heightened ability to determine intent in others and integrate sensory information into one’s own emotional schema relates to flexibly shifting attention towards the target of interest (either “self” or “other”). In sum, our data suggest the IFO may serve as an internal/external attention modulator and thus may play a critical role in switching attention from one’s own body sensations (“self”; IA) to the other’s (Affective and/or Cognitive empathy).

Our study’s findings should be considered along with its limitations. The definition of rsBOLD has been inconsistent across previous studies (e.g., amplitude, variance, standard deviation, mean squared successive difference; for a review, see Garrett et al., 2013), with a considerable range in the methodology used to derive them. Therefore, the implementation of rsBOLD as a consistently used neuroimaging measure will require increased efforts toward methodological standardization. It is also important to note that due to the nature of the analyses used, the findings of this study do not represent causal relationships. That is, the results represent a correlational relationship between a questionnaire-based measure of IA or empathy and rsFC and/or rsBOLD. Our sample was unfortunately not large enough for a gender-specific analysis, as evidence suggests there are differences in the capacity for empathy between males and females (Christov-Moore et al., 2014). Future research should be conducted in this regard. Similarly, in using an undergraduate sample, the generalizability of these findings is limited.

In conclusion, the current research provides novel information about the relationship between IA and empathy. In contrast to previous studies that used task-based fMRI to assess the neurobiology of these two constructs separately, we used a data-driven resting-state approach to test whether distinct empathy facets share network characteristics (rsFC/rsBOLD) with IA. We demonstrate a bidirectional relationship between empathy and IA, depending on the type of empathy investigated. Specifically, Affective empathy and IA share rsFC and rsBOLD, while Cognitive empathy and IA only share rsBOLD. Concerning Affective empathy, increased vicarious emotional experience and decreased IA were associated with increased rsFC within the rIFO of a larger attention network; while increased IA and reduced Affective Empathy were related to increased network flexibility within an internal sensation network. Concerning Cognitive empathy, perspective-taking ability, and a sense of mind-body connectedness related to increased communication between brain regions subserving a mentalizing network. We also suggest the role of the IFO as an internal/external attention modulator that may play a critical role in switching attention from one’s own body sensations (IA) to another’s (Affective and/or Cognitive empathy). Overall, we show that the ability to feel and understand another’s emotional state is related to one’s awareness of internal body changes and that this relationship is reflected in the brain’s intrinsic neuroarchitecture. Methodologically, this work highlights the importance of utilizing rsBOLD alongside rsFC as an important complementary route into understanding neurological phenomena. Our results hold promise in aiding diagnosis of clinical disorders characterized by IA and empathy deficits such as the autism spectrum disorders (ASD), where participants may be unable to complete tasks or questionnaires due to the severity of their symptoms.

## Data Availability Statement

The datasets presented in this study can be downloaded from: https://interoceptionempathydataset.s3.us-east-2.amazonaws.com/InteroceptionEmpathy_Data.zip.

## Ethics Statement

The studies involving human participants were reviewed and approved by University of Louisville IRB. The patients/participants provided their written informed consent to participate in this study.

## Author Contributions

TS: data curation, investigation, project administration, formal analysis, methodology, visualization, and writing—original draft. BD: resources, supervision, and writing—review and editing. All authors contributed to the article and approved the submitted version.

## Conflict of Interest

The authors declare that the research was conducted in the absence of any commercial or financial relationships that could be construed as a potential conflict of interest.
